# Ischemic Colitis Induced by Concurrent Non-steroidal Anti-inflammatory Drug (NSAID) and Corticosteroid Use for Back Pain

**DOI:** 10.7759/cureus.90548

**Published:** 2025-08-20

**Authors:** Yusuf Almascati, Ali Alhumaiqani, Bernard Wen, Talal Alarayedh

**Affiliations:** 1 Internal Medicine, King Hamad American Mission Hospital, A'ali, BHR; 2 Internal Medicine, Royal College of Surgeons in Ireland, Busaiteen, BHR

**Keywords:** corticosteroid side effects, drug-induced colitis, gastrointestinal ischemia, ischemic colitis, nonsteroidal anti-inflammatory drugs (nsaids)

## Abstract

Ischemic colitis is a multifactorial condition typically seen in older adults with risk factors for vascular disease and hypoperfusion, but it can also occur in healthier individuals due to medication-induced mucosal injury. We discuss a case of a 55-year-old male who developed right-sided ischemic colitis following two weeks of high-dose nonsteroidal anti-inflammatory drug (NSAID) use, combined with systemic corticosteroids for back pain. He presented with severe abdominal pain and bright red rectal bleeding, with labs suggestive of inflammation, initially raising the concern for gastritis and infectious colitis. Imaging showed nonspecific, acute mucosal inflammation, but endoscopic evaluation with biopsies confirmed the diagnosis of ischemic colitis, rather than infectious colitis. Conservative management with bowel rest, IV fluids, pain medications, and antibiotics led to a full recovery, with complete healing of mucosal ischemia on follow-up colonoscopy. This case demonstrates the potential for drug-induced ischemic colitis in patients without classic causes and focuses on the importance of careful medication review in patients presenting with acute colitis.

## Introduction

Ischemic colitis is defined as an inflammatory condition of the colon due to a transient or persistent reduction in blood flow, leading to ischemia and mucosal injury [1]. The splenic flexure and sigmoid colon are the most common anatomic locations of ischemic colitis due to their dependence on collateral circulation. These areas are often termed “watershed areas” since they are supplied only by the small, terminal branches of the superior and inferior mesenteric arteries, as well as the terminal branches of the superior rectal and inferior mesenteric arteries [2]. Any hypoperfusion can lead to disproportionate loss of blood flow, and therefore ischemia, to these watershed areas, due to their lack of direct vascular supply.

Typical causes of hypoperfusion can include hypotension, heart failure, vasoconstriction, or thromboembolism, all of which compromise vascular health by reducing effective colonic perfusion. In addition to vascular disease and systemic hypoperfusion, ischemic colitis may also be drug-induced by medications such as nonsteroidal anti-inflammatory drugs (NSAIDs), which can compromise mucosal defenses and precipitate ischemia. After the initial insult, ischemic damage may also be worsened by reperfusion injury [3], where oxidative stress and inflammatory cells amplify ulceration and necrosis.

Although ischemic colitis is the most common form of intestinal ischemia [4], its resemblance to infectious or inflammatory colitis often delays diagnosis. Endoscopy may be required, showing patchy ulceration, cyanotic mucosa, and rectal sparing [5]. Delayed diagnosis of ischemic colitis can lead to progressive necrosis, bowel perforation, or sepsis. In this case report, we discuss a patient who presented with less common risk factors for ischemic colitis, which made the diagnostic process more complicated.

## Case presentation

A 55-year-old male with hypertension, gastroesophageal reflux disease, and long-standing tobacco smoking presented with severe right-sided abdominal pain and multiple episodes of fresh rectal bleeding. His symptoms persisted despite the use of antacids and proton pump inhibitors.

History revealed a recent two-week course of high-dose NSAIDs to manage acute back pain due to a newly diagnosed lumbar disc prolapse. He was also prescribed a six-day course of oral prednisolone (40 mg twice daily), in addition to diclofenac, pregabalin, and pantoprazole. He had been chronically taking low-dose aspirin for over 15 years as primary prevention for atherosclerotic cardiovascular events. He denied any personal or family history of inflammatory bowel disease or gastrointestinal bleeding.

On examination, he was alert but dehydrated and afebrile, blood pressure was high at 186/100 mmHg, he was tachycardic at 114 beats per minute, and oxygen saturation was 97% on room air. The patient reported severe abdominal pain, yet physical examination revealed only mild epigastric tenderness, a classic discrepancy seen in ischemic colitis. Digital rectal examination was refused by the patient.

Abnormal blood tests included an elevated white blood cell (WBC) count of 14.3 × 10^9^/L, an elevated C-reactive protein (CRP) of 192 mg/L, and an elevated lactate of 3.65 mmol/L. Remaining blood tests, including electrolytes, creatinine, and coagulation studies, were within normal limits. Stool testing showed numerous red and white blood cells on microscopy, and an elevated fecal calprotectin at 663 µg/g. The relevant laboratory results are summarized in Table 1.

**Table 1:** Key laboratory findings: admission and discharge WBC: White blood cell count; CRP: C-reactive protein; HPF: High-power field; RBC: Red blood cell count

Parameter	On Admission	At Discharge	Reference Range
WBC	16.0 × 10⁹/L	7.3 × 10⁹/L	4.0-11.0 × 10⁹/L
Hemoglobin	16.3 g/dL	13.5 g/dL	13.5-17.5 g/dL
CRP	192 mg/L	58 mg/L	<5 mg/L
Platelet Count	298 × 10⁹/L	197 × 10⁹/L	150-400 × 10⁹/L
Urea	9.5 mmol/L	6.8 mmol/L	2.5-7.1 mmol/L
Creatinine	98 µmol/L	81 µmol/L	60-110 µmol/L
Lactate	3.65 mmol/L	1.09 mmol/L	0.5-2.2 mmol/L
Fecal Calprotectin	663 µg/g	–	<50 µg/g
Stool RBCs	Numerous	–	Not normally present
Stool WBCs	40–50/HPF	–	Not normally present

He was admitted for suspected NSAID and steroid-related gastrointestinal bleeding versus an infectious colitis. NSAIDs, steroids, and aspirin were immediately discontinued, and he was started on intravenous fluids, a proton pump inhibitor infusion, and empiric antibiotics. An urgent esophagogastroduodenoscopy (EGD) revealed severe ulcerative esophagitis, gastric antral erosions, but no source of active gastrointestinal bleeding, nor a clear cause of his severe abdominal pain. A contrast-enhanced CT of the abdomen showed right-sided colonic wall thickening and edema, which was indicative of acute mucosal inflammation, but the differentiation between infectious and ischemic colitis remained unclear.

**Figure 1:**
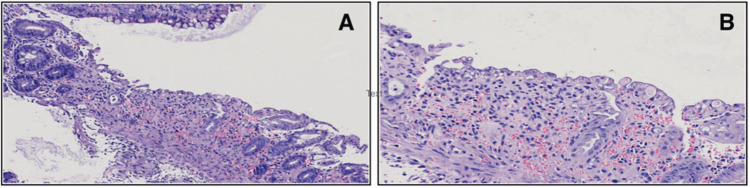
Colonic biopsy findings consistent with ischemic colitis (A) Low-power magnification (10×) showing surface epithelial attenuation, crypt dropout, and lamina propria hemorrhage. (B) Higher magnification (20×), revealing regenerative epithelial changes, mild neutrophilic infiltration, and focal small vessel necrosis.

At that point, colonoscopy was considered to clarify the diagnosis, but ultimately deferred to allow mucosal inflammation to settle. During this period, the patient remained hemodynamically stable and gradually improved with ongoing bowel rest, intravenous fluids, and broad-spectrum antibiotics. Once his condition stabilized, a colonoscopy was performed, which showed patchy, friable, and ulcerated mucosa extending from the cecum to the splenic flexure of the colon, with an abrupt transition to normal-appearing colonic mucosa distally. Colonic biopsies showed surface epithelial attenuation, crypt dropout, lamina propria hemorrhage, and focal small vessel necrosis, all findings consistent with ischemic injury (Figure 1), confirming the final diagnosis of ischemic colitis.

**Figure 2:**
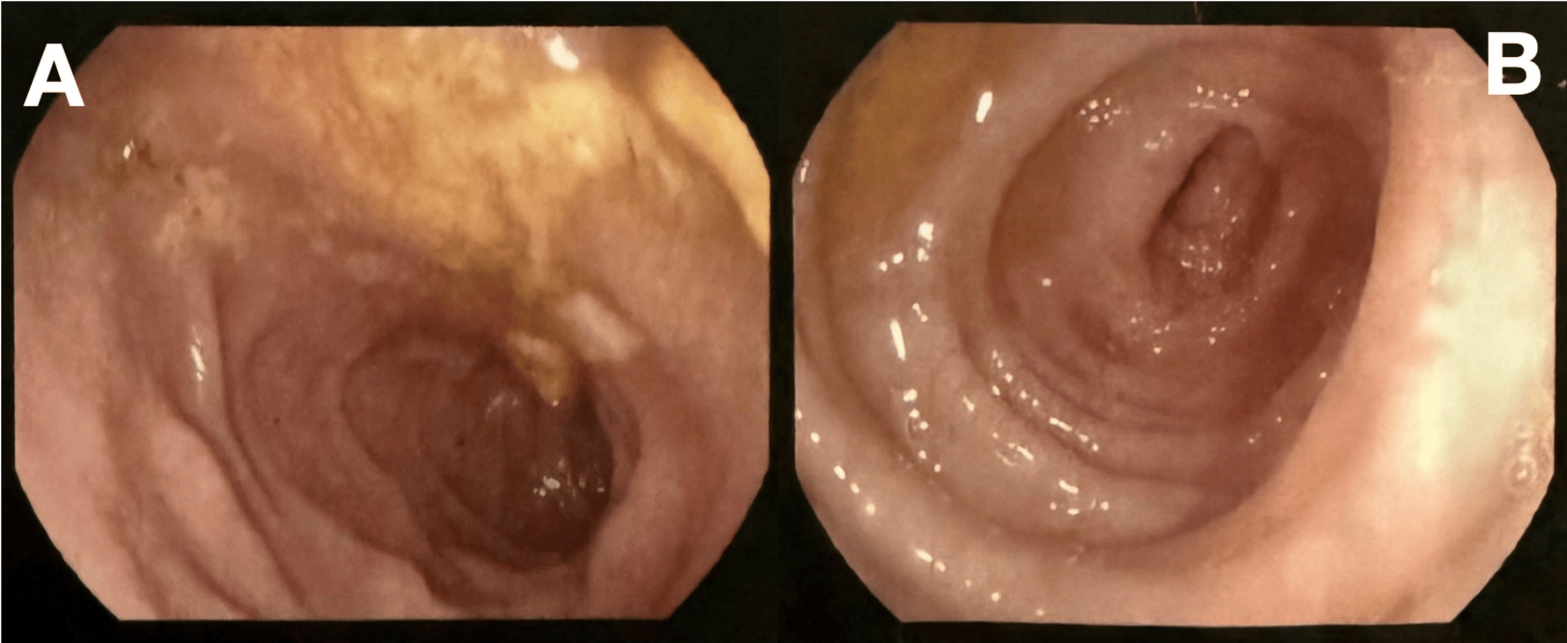
Esophagogastroduodenoscopy (EGD) before and after treatment (A) Initial EGD: severe ulcerative esophagitis and antral erosions. (B) Follow-up EGD: mucosal healing with resolution of esophagitis and gastritis.

**Figure 3:**
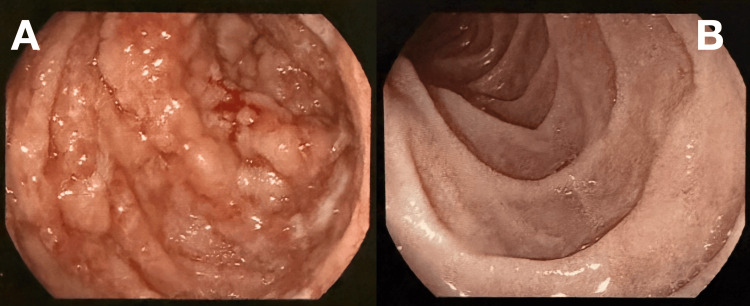
Colonoscopy before and after treatment (A) Initial colonoscopy: friable, ulcerated, and hemorrhagic mucosa from the cecum to the splenic flexure. (B) Follow-up colonoscopy: complete mucosal healing and restoration of normal colonic architecture.

With ongoing supportive care, the patient’s abdominal pain and rectal bleeding completely resolved, and his markers of inflammation (WBC and CRP) continued to trend downward. He was transitioned to oral antibiotics and discharged with strict instructions to avoid further use of NSAIDs and systemic corticosteroids. A follow-up EGD and colonoscopy performed two months later showed complete mucosal healing of both his esophagus and colon (Table 2), with histopathologic resolution of the previously seen colonic ischemia (Figures 2-3).

**Table 2:** Timeline of endoscopic evaluations and findings EGD: Esophagogastroduodenoscopy; GI: Gastrointestinal

Hospital Day	Procedure	Key Findings	Interpretation
Day 2	Esophagogastroduodenoscopy (EGD)	Severe ulcerative esophagitis and antral erosions. No active upper gastrointestinal bleeding	NSAID-induced upper GI mucosal injury
Day 5	Colonoscopy	Friable, ulcerated, hemorrhagic mucosa from cecum to splenic flexure. Abrupt transition to uninvolved mucosa	Acute right sided ischemic colitis
Day 63	Follow-up colonoscopy	Normal mucosal appearance with complete resolution of previous inflammation and ulceration	Full mucosal recovery post-treatment

## Discussion

Ischemic colitis is the most common form of gastrointestinal ischemia, typically affecting older adults over the age of 60 years with atherosclerotic disease or systemic hypotension. However, younger individuals without classic vascular risk factors may also develop ischemic colitis, particularly in the setting of medication-induced mucosal injury. Recognizing this variant of ischemic colitis requires astute clinical consideration, as it is often misdiagnosed due to its atypical risk profile [6]. In our case, a previously healthy male presented with acute right lower quadrant abdominal pain and hematochezia following two weeks of high-dose NSAID use combined with systemic corticosteroids. While the initial concern was focused on other, more commonly considered diagnoses such as infectious colitis and gastritis, the imaging, endoscopy, and histology findings pointed instead to ischemic colitis, particularly with the pattern of mucosal injury seen, where there was an abrupt transition from ulcerated to normal colonic tissue.

In the absence of hypotension or vascular thrombosis, the typical causes of ischemic colitis, the patient’s recent use of NSAIDs and steroids and tobacco smoking emerged as the likely causes. In a recent study of more than 1,500 ischemic colitis cases, Twohig et al. reported that, aside from established risk factors, certain medications (beta-blockers) and pro-inflammatory systemic diseases (including vasculitis, rheumatoid arthritis, and malignancy) were also significantly associated with ischemic colitis, indicating that they may play a role in patients without classic comorbidities [7]. NSAIDs specifically are known to compromise colonic perfusion by inhibiting prostaglandin synthesis, impairing epithelial defenses, and promoting vasoconstriction [8]. A study by Xu et al. described NSAID-associated ischemic colitis in patients without systemic hypotension or overt atherosclerotic disease, supporting the role of localized drug-induced perfusion compromise [9]. Similarly, corticosteroid use has been associated with delayed mucosal healing and increased susceptibility to injury in pre-existing ischemic and inflammatory bowel conditions [10]. While upper gastrointestinal complications due to NSAIDs and steroids, such as gastritis and peptic ulcers, are well-documented, their role in lower gastrointestinal complications, such as ischemia, is underrecognized. In our patient, ischemic colitis may have been further exacerbated by smoking tobacco, which can impair microvascular flow. The combination of these medication-induced insults likely overwhelmed his mucosal defenses and precipitated ischemia in a susceptible watershed area of the colon.

Unusually, this patient’s area of abdominal pain and ischemia was in the right-sided colon, which is less known as a potential watershed area of the colon. Typically, the watershed areas are taught to include the splenic flexure and the sigmoid colon, both on the left side of the colon; therefore, the usual illness script of ischemic colitis highlights left-sided abdominal pain. However, the right colon does have an area that lies at the junction of the superior mesenteric artery and marginal artery [11], creating another watershed area with limited collateral blood flow that is susceptible to hypoperfusion and vasoconstrictive injury. Right-sided ischemic colitis can actually be more severe, due to its atypical presentation, leading to delayed diagnosis and an increased risk of developing into full-thickness necrosis. In a population-based cohort study, Montoro et al. found that right-sided colonic ischemia was associated with increased morbidity and a higher likelihood of surgical interventions [12]. Fortunately, the clinical course in our patient was favorable, due to the vigilance of the gastroenterologist who recommended ongoing consideration of ischemic colitis, which eventually led to endoscopic confirmation of colonic ischemia.

Uniquely, our case serves as a valuable example of how inappropriate medication usage can lead to serious complications. Inappropriate NSAID use remains a leading contributor to drug-induced gastrointestinal injury, often under-appreciated in younger or otherwise healthy populations [13]. In younger patients, the absence of classic vascular risk factors is often misattributed as low-risk, which may lead to more frequent NSAID and steroid prescriptions, and may also delay the recognition of serious gastrointestinal adverse effects. Our patient was prescribed both NSAIDs and systemic steroids without any documented medication review or personalized assessment of gastrointestinal risk. As NSAID use remains prevalent in outpatient settings, especially for common problems such as musculoskeletal pain and upper respiratory tract infections, physicians should be more aware of its full range of gastrointestinal complications, particularly ischemic colitis. Although there is usually more caution before prescribing systemic steroids, there should still be further care in reviewing a patient’s full list of medications, including over-the-counter ones, for possible other medications such as NSAIDs that may increase the risk of gastrointestinal complications when combined with steroids.

## Conclusions

Ischemic colitis is a gastrointestinal emergency with a broad range of causes, often underrecognized in its early stages when symptoms and radiologic features mimic other forms of colitis. Right-sided ischemic colitis is even more uncommon, but it should be considered even in patients without classic hypoperfusion states, particularly when subtle risk factors such as NSAID or steroid use are present. For our patient, an early endoscopic approach led to a definitive diagnosis with histopathologic confirmation. Withdrawal of offending agents, bowel rest, and empiric antibiotics allowed for a full recovery without complications or surgical intervention.

This case features several key clinical lessons. Firstly, ischemic colitis should remain on the list of differential diagnoses in patients presenting with symptoms of acute colitis, even in younger patients without obvious risk factors. Secondly, a thorough medication review should be mandatory for any patient presenting with acute colitis, as it may reveal overlooked etiologies. Thirdly, before prescribing new medications, risks versus benefits and potential interactions must be carefully evaluated. Clinician awareness of medication-induced ischemic colitis is essential for early recognition, appropriate workup, and safe prescribing practices. Future efforts may involve standardized risk stratification prior to starting potentially injurious medications.
